# It’s complicated: staff experiences of academic integrity at a Mauritian university

**DOI:** 10.12688/f1000research.167245.1

**Published:** 2025-08-08

**Authors:** Annemarie Davis

**Affiliations:** 1 Business Management, University of South Africa College of Economic and Management Sciences, Pretoria, Gauteng, 0003, South Africa

**Keywords:** academic integrity; qualitative research; Mauritius; professional staff; ethics in education

## Abstract

**Background:**

Academic integrity is presented as a complex, multidimensional concept that involves all members of an educational institution. However, non-teaching staff members are often excluded from research on academic integrity. I engaged in professional support alongside academic staff to explore how academic integrity is understood within the Mauritius branch of a UK higher education institution.

**Methods:**

I adopted a qualitative approach to engage those tasked with implementing academic integrity to examine how they understood and practised it. Data were gathered through face-to-face focus-group interviews, which were translated into themes.

**Results:**

This article identified the factors that make academic integrity challenging and presents recommendations for practitioners and researchers.

**Conclusions:**

More energy should be invested in the processes that build cultures of integrity rather than focusing on the opposite, such as cheating, plagiarism, and misconduct. Academic integrity is ultimately a positive values-driven approach to learning and a skill that can be developed and strengthened.

## Introduction

More than three decades ago, Moffatt (1990) claimed that cheating comes as naturally as breathing does. Some may argue that this is a very cynical view and that much has been done in institutions of higher learning over the last 35 years. The use of honesty statements, honor codes, decrees of integrity, and tools to detect plagiarism has increased, with associated contributions to the bodies of knowledge on academic integrity, academic misconduct, and ethical standards in higher education assessment. However, cheating remains a major problem in higher education, now enabled through technological advancements and tools, the widespread adoption of large language models, the prevalence of contract cheating, online assessments, and the perceived acceptance of a cheating culture. Issues of academic integrity extend beyond the classroom to the institution as a whole ( Whitley & Keith-Spiegel, 2010). Recently, Mahmud and Ali (2023) described a shift in the literature on academic integrity through bibliometric analysis spanning two decades. Their research found a continued focus on plagiarism and academic misconduct, with a definite move towards academic integrity and contract cheating in the second decade. Mahmud and Ali (2023) also found that research continues to be segmented, with researchers tending to use the lens of either “misconduct,” viewed as behavior lacking honesty, or “integrity,” viewed as behavior upholding values, including honesty. Goddiksen et al. (2023) referred to the gray zones between cheating and good practice, and found that students have a poor understanding of concepts such as plagiarism and falsification.

A foundational concept that shaped this research project was that academic integrity is a multi-stakeholder, multi-faceted concept that applies to everyone within an educational institution ( Peters, 2018). However, non-teaching staff members are rarely included in academic integrity conversations and research. Subsequently, Davis (2023) called for research among professional support staff as important roleplayers in the practice of academic integrity.

This article reports research conducted among professional support staff and academic staff members at the Mauritius branch of a UK institution of higher learning. Specifically, I set out to answer research questions pertaining to how academic integrity is understood at the institution and to identify the factors that complicate academic integrity practices within the selected institution. Through engagement with professional and academic staff, I present recommendations for policymakers and suggest areas for future research.

As indicated by the title, academic integrity is complex. As a practicing academic and researcher interested in academic management, I explored the lived experiences of academic and professional staff regarding how academic integrity is practiced within a selected university. Building on earlier research that confirmed the need to foster a greater understanding of academic integrity in universities ( Davis, 2023), this study presents findings on the factors that complicate academic integrity and affirms the diverse understanding of academic integrity.

### The researcher’s position

Aligned with the nature of qualitative research, specifically the descriptive phenomenological approach employed in this study and the associated practice of reflexive bracketing, I offer a brief description of my prior experiences and connections to the research topic ( Squires, 2023). I am an academic staff member at the largest single-mode distance education institution in South Africa, which caters annually to more than 300 000 students. Within the institution, I form a part of the teaching team for a module with more than 2 000 enrollments per year.

At the time of data gathering, my personal experience with academic misconduct was an extreme frustration and administrative burden. Additionally, the data gathered in the Mauritius case institution represents the second phase of a broader research project on academic integrity in higher education institutions. At the time of the focus group sessions, I had already completed the analysis and reporting in the first phase of my research. Some of the existing premises that were fresh in my memory during the interviews pertained to diverse perspectives on what constitutes academic integrity and the contradictory realities faced by academics in its practice.

In my personal capacity, my values, background, and cultural supposition are geared towards enhancing and adhering to the highest standards of integrity and ethical practices. I find myself unsympathetic towards academic transgressors and try to distance myself from academic practices that raise questions about standards, ethics, and integrity. At the same time, through my prior research, I recognize that there are different interpretations of academic integrity and how it is practiced in contradictory contexts ( Davis, 2023).

### A review of the literature on academic integrity

Despite calls for a unifying framework to bring issues of ethical and unethical behavior together, academic integrity is still viewed through the lens of ‘misconduct’ or as behavior lacking honesty or integrity ( Mahmud & Ali, 2023). While some may argue that progress has been made in reframing academic integrity as an academic skill ( Eaton, 2024; Davis, 2023; Yeo, 2023; Sotiriadou, Logan & Guest, 2020), questions remain regarding the potentially ambiguous nature of how academic integrity is perceived in practice. One author even argues that there are so many contradictions in practice that the diverse perspectives on what constitutes academic integrity are not unexpected ( Davis, 2023).

Although it is not my intention to discount these views on the diverse perspectives and associations with misconduct, I need to offer a definition of academic integrity as the foundational concept that informed this research. I offer definitions of academic integrity from the literature and then focus on how it is defined within institutional policies and documents. The institutional definition is the starting point for focus group conversations held by professional support staff and academic staff.

### Defining academic integrity

As previously noted, there is no singular or universally accepted definition of academic integrity ( Eaton, 2024). An EU-wide exploration of academic integrity policies and systems across 27 countries confirmed varied views on academic integrity. Using an academic integrity maturity model, Foltýnek and Glendinning (2015) found a stark divide between Western and Eastern EU nations, and emphasized the significant variability in academic integrity policies across different regions.

The European Network for Academic Integrity declares academic integrity as compliance with ethical and professional principles, standards, practices, and a consistent system of values that serves as guidance for making decisions and taking actions in education, research, and scholarship ( Tauginienė et al., 2018, p. 9). The International Centre of Academic Integrity in Canada defines academic integrity as a commitment to honesty, trust, fairness, respect, and responsibility. By summarizing the main results of previous articles that discussed the nature of academic practices, Aronson (2024) presented academic integrity as uncorrupted moral virtue in relation to truth, uprightness, and sincerity in the pursuit of research, education, and scholarship (p. 1). Academic integrity is viewed as an ethical scholarship that avoids academic dishonesty ( Bretag, 2012, 2013).

The Bretag (2012) academic integrity policy model provides a robust basis for analysis and policy enhancement, and has been used in several studies. However, Perkins and Roe (2024) found that no studies have identified policies that satisfy all required elements of the Bretag model.

While recognizing the divide between Western and Eastern EU nations ( Foltýnek & Glendinning, 2015), the selected case institution is a UK university operating in the SADC region (Mauritius). In the context of the case institution, academic integrity is described as a set of learned skills with honesty, fairness, and respect for others and their work at the core. I am affiliated with a South African university and present the definition from my home institution here in an attempt at transparency and sharing the foundation of my lens as practiced in my daily activities as a lecturer, scholar, and researcher. Academic integrity is defined as meaningful and concerted efforts to ensure concern for human dignity, honesty, trust, fairness, truthfulness, accuracy, respect, and responsibility in teaching, research, and community engagement ( University of South Africa, 2017).

A range of keywords is closely linked to academic integrity. Perkins and Roe (2024) analyzed academic integrity policies by adopting corpus linguistic techniques and supplementing them with inductive content analysis. The most common keywords identified in their analysis included plagiarism, misconduct, integrity, cheating, dishonesty, discipline, and turnitin. The findings of the Perkins and Roe (2024) analysis are not surprising, as these keywords also appear in the case institution’s policy as well as in that of my own institution. Therefore, it is not surprising that academic integrity continues to be viewed through the lens of misconduct when one considers the content of various policies. However, understanding how practitioners view academic integrity in practical settings may offer insights into practices that shape academic integrity.

### Research proposition

In support of East and Donnelly (2012), I concur that more systematic approaches are needed to teach both students and academics about academic integrity and to provide resources that promote academic integrity as an educational opportunity, rather than focusing on punishment. The current research proposition is that gaining insight into how academic integrity is perceived by professional support staff and academics offers a deeper understanding of how those tasked with implementing it conceptualize and practice it. These insights may be especially valuable given that the case study institution is a UK institution operating within a developing economy. Research on how academic integrity is perceived in a branch campus of a UK university located in an African country is therefore likely to strengthen the understanding of the concept and play a vital role in driving change and preparing future academics, researchers, administrators, and graduates to lead the universities of tomorrow.

### Methodology

Exploring the perceptions and practices of academic integrity among professional support staff and academics requires a descriptive phenomenological approach. According to Husserl and Moran (2012), descriptive phenomenology aims to distinguish, understand, and clarify the essential features of a defined phenomenon from the perspective of the persons directly involved. This approach, enabled through face-to-face focus group interviews, provided a robust way to tease out and reveal feelings and experiences by capturing small, time-bound human understanding, which I then translated into themes ( Sinfield et al., 2023, p. 2).

Ethical clearance for the research was granted by my institution through the recognized committee (2022_CRERC_028_FA) along with permission from the case institution in Mauritius. Data were gathered in October 2023 through two research focus group sessions. The first focus group was conducted with academic staff members and the second focus group was conducted with professional support staff. Overall, 11 participants provided rich descriptions in response to the focus-group question guide. The academic staff group comprised part-time faculty members across disciplines, while the professional group included members from the registrar’s office, admission office, and student liaison office. Focus group discussions were audio-recorded and transcribed, resulting in 52 pages of transcriptions. I opted for verbatim transcriptions to ensure that I had captured all potentially relevant data. My analysis process is oriented towards lived experience and the interpretation of the text ( Creswell & Poth, 2016; Fossey, Harvey, McDermott & Davidson, 2002), focusing on what, how, and why of experiences related to academic integrity.

Before commencing the focus group sessions, I familiarized myself with the various rules and regulations pertaining to academic integrity and misconduct shared with me by the case institution. I also shared the participant information sheet with participants before the focus group sessions. The participant information sheet forms part of the research ethics procedures at my institution, but it also contributes to fair research practices and informed participant consent. All participants consented to participate in the research through a written statement signed and submitted to me for record keeping. The participant information sheet offered an overview of the topic, purpose of the research, assurance of anonymity, and an indication of how the findings would be shared. Participant recruitment was handled through the university registrar’s office, which sent out an invitation on my behalf and arranged the date, time, and venue for the focus group sessions. I had five participants in the professional staff session, representing professional staff at the director, manager, and administrative level. The academic staff focus group included six participants from all faculty members.

I led the discussions in both focus group sessions and, at that time, had already identified potential themes. Yet, as a qualitative researcher, I intentionally suspended judgments and allowed time to pass between the sessions and reading of the transcripts. In alignment with Tisdell, Merriam and Stuckey-Peyrot (2025), I adopt a bracketing approach to temporarily set aside biases, prejudices, and assumptions. This process of “waiting in the wings” ( Sinfield, Goldspink & Wilson, 2023, p. 1) to reduce bias was intentional.

Given the descriptive phenomenological orientation, I considered the methodological process suggested by Swanson-Kauffman and Schonwald (1988) of bracketing, analyzing, intuiting, and describing. While conducting the research, I found that these steps merged with each other. Through the conscious act of bracketing, I was able to push my assumptions and thoughts aside and become more open to the lifeworld of the participants at the case institution. However, bracketing was not a once-off task; I revisited my own assumptions regularly, which required deliberate efforts to push aside invasive thoughts, prior experiences, and own practices.

Invariably, the data analysis process took longer because of both the planned delay in analyzing the data and my revision of the bracketing process. The analysis began with the first reading of the transcripts. As indicated earlier, I allowed time to lapse and mindfully distance myself from the data. Before commencing with the analysis, I familiarized myself with recent literature on academic integrity and revisited my bracketing. I also accessed the audio recordings to listen to the focus group discussions and ground my attention to the topic. Simultaneously, I read the transcripts to ensure my accuracy. Following this, I printed the transcripts to read and re-read, making preliminary notes on the printed pages. I used colored pens, mind-map techniques, and post-it notes to organize my preliminary thoughts on the themes. I have highlighted significant statements that I would later use to support my assertions. The process of intuiting took shape through the identification of these significant statements and drawing of mind maps to categorize the data. Over a period of eight days, I constantly revisited the raw data to enhance my sense-making and conceptualize the themes that would inform my analysis. While this may seem like a prolonged process, I believe it has strengthened my application of descriptive phenomenology.

While working with the data, I also reviewed documents shared by the case institution, including official academic regulations (focusing on the section addressing academic integrity and misconduct) and two annual reports (2021 and 2022) prepared by the academic misconduct team. Annual reports were used to provide the contextual richness.

My data analysis was a manual process supplemented with the use of the Atlas.ti program, which assisted in sorting the data and retrieving verbatim quotes to support the themes. The use of Atlas.ti also contributed to mapping my sense-making and enhancing the transparency of the research process.

## Discussion

### Theme 1: Understanding of academic integrity

This theme was crafted from the descriptions of the participants when they were asked to explain what academic integrity meant to them. Not surprisingly, the descriptions were mostly negatively framed; that is, not to do something. The negative framing of academic integrity is not uncommon, as reported by Macfarlane, Zhang, and Pun (2014) and confirmed earlier in this article.

What is interesting is the direct association between the concept of “integrity” and “misconduct,” which represents the first theme identified from the data. From the participants’ descriptions, there appears to be a contrast between academic integrity and misconduct. Table 1 depicts the words used by the participants, positioned under the themes of academic integrity and misconduct. The letters in brackets following the descriptors indicate the participant group: A for academic staff and P for professional support staff.

**Table 1.** Descriptors used by participants.

Academic integrity	Academic misconduct
Being honest (P)	Cheating (A)
Respect (P)	Collusion (A)
A guarantee of what we offer (P)	Contract cheating (A)
Ethical practice (A)	Academic bad practice (P)
Referencing (A)	
Not stealing (A)	

Interestingly, within the research focus group with the academic staff, the conversation shifted towards activities outside of formal assessment, such as disrupting classes, misbehaving in class, or fighting on campus. Upon probing, one participant said,

Academic integrity would be about studies. Whatever is connected to their, for example, referencing. However, academic misconduct is much thicker. Therefore, for me, there are some differences between the two. The big set would be academic misconduct and the subset would be academic integrity. (Participant 2)

Reflecting on the case institution’s policy ( Middlesex University Mauritius, 2023, p. 64) the section on infringement of assessment regulations/academic misconduct refers to the contraventions of regulations through negligence, foolishness, or deliberate intent. It also offers 15 examples of academic misconduct, including transgressing examination room rules, presenting work as one’s own that is wholly or partially the work of another, offering a bribe to invigilators or academic or administrative staff, presenting unauthorized groupwork as the work of a single candidate, and using any form of unfair or dishonest assessment practice. The policy then outlined the recommended procedures and guidelines for penalties.

I also asked how the academic staff members managed their academic integrity. A strong message emerged from the academic focus group: their role is not to “manage” it, but rather to remind students about academic integrity, to explain it using examples, and to offer support in developing it as a skill. One participant explained the value of using a Turnitin report to show students their similarity reports.

My findings concur with Löfström, Trotman, Furnari, and Shephard (2015), who found, in research among academics in New Zealand and Finland, that while academics were united on the importance of academic integrity, they were not of one mind about what it is and whose responsibility it is to teach it.

After establishing the staff’s views on academic integrity, I asked them how they thought the students understood the concept. The following were notable: “I think we try our best to explain it, but I do not think they register it properly” (Participant 4).

There appeared to be agreement that despite the efforts of staff, the institution, and the policy, students still engage in academic misconduct. One participant expressed her view that some students are: “… completely oblivious to what might happen or how that will have an impact on their grades if they are caught” (Participant 4).

While there have been efforts to promote academic integrity as an academic skill, it seems the stick (in the proverbial carrot and stick approach) may continue to be used. One participant stated, The students actually only interrogate the documents once they have been accused of misconduct (Academic Participant 3).

Reflecting on this section about how academic integrity is understood, I accept that it is really complicated. Nevertheless, I fully concur with Bretag ( Peters, 2018) that it is critical that every member of the academic community takes responsibility for academic integrity within their specific spheres.

### Theme 2: Factors complicating academic integrity

Within this theme, I aimed to understand the factors that complicated academic integrity practices in the two groups. I did not specifically limit the responses to the management or administration of academic integrity; rather, I allowed the conversations to take a natural flow and only prompted them for clarity.

The identified factors are grouped under systems and people.

*Systems*

Technology and resources were included within the system theme. Technology use has both positive and negative associations. For example, technological developments have been blamed for the breach of academic integrity. The use of Artificial Intelligence (AI) in academia has attracted increasing attention. Although there are benefits to tools such as ChatGPT, concerns remain regarding academic integrity and plagiarism. When asked for examples of academic integrity breaches, one participant stated:

Last year, ChatGPT was launched in November. I think it was the 25th of November. By the 10th of December we had the first case. It was not even available in Mauritius, and this was our first case. (Professional Participant 5)

There appears to be support for students using AI in their studies because, as an academic participant explained,

… the world of work will probably also make use of tools, such as ChatGPT. However, there was a warning: Using it as a tool is not bad, but here they are just using it to submit the assignment, and they do not even understand what is being written there. (Participant 1)

A positive association with technological developments also emerged in relation to plagiarism detection tools. On a more formative note, I appreciate the descriptions of how these tools are used in teaching. One academic participant explained how she uses Turnitin reports from former students as examples to teach current students what constitutes acceptable and unacceptable work. Among professional support staff groups, the value of Turnitin was also affirmed and linked to policy enforcement. One participant noted:

However, even the students can actually use Turnitin as a tool to improve their work because if they submit, they get the opportunity to submit more than once and they will be able to identify if they do not paraphrase well; for example, they get an opportunity to do that better. Thus, it is a tool that students can use for their advantage. (Professional Participant 1)

Technology has also enabled easier communication – but I heard about instances where targeted messages and social media are used to ‘lure’ students into academic misconduct practices. For example, in both groups, participants described situations where students received messages such as “I will write your essay for you. Click here. Pay this amount.” (Participant 3).

Both groups confirmed that such offers may be very tempting when students are “stressed out, overworked.” Within my own institution, I have also experienced an overwhelming drive from contract cheaters to engage students, which reminded me of Peters (2018), who stated that the ways in which students cheat have changed dramatically in recent times ( Peters, 2018). Commercial cheating sites deliberately target students who are under time pressure. These contracted cheating options offer a way to distract students from effortful learning.

Resource limitations are also presented as challenges. In the academic focus group, resource constraints were highlighted when dealing with cases of academic misconduct. If a lecturer has to manage multiple cases simultaneously, it can become overwhelming, leading to the inconsistent handling of such cases.

*People*

The factors within this theme include the students, faculty, and professionals. The “people” factor encompasses actions, perceptions, and lived experiences. As described by the participants in both focus groups, when academic integrity is enforced inconsistently, students become confused about what is acceptable.

We need to apply a policy across everyone. If a student knows that that the lecturer allows that to go through, this lecturer does not. That lecturer is not going to be unpopular. A lecturer that does not allow it is unpopular. (Professional Participant 5)

Yet, within both groups there was affirmation that “we’ve got that right.” What seems to complicate consistent application is the administrative burden associated with investigating and reporting incidents. Academic Participant 2 referred to the realities of class sizes and the effort involved in reporting misconduct.

It is very different when you have a class of 17 than when you have a class of 70. …the teachers being human beings as well, they need to make rational decisions about where I shall put my time and effort. So, sometimes it’s much easier when you manage a class of 70 to give a bit of slap on the wrists of the student, literally, and say, “This is not acceptable, you should submit something else” as compared to when you have a class of 17 where you can actually fill in the paperwork. (Participant 2)

One academic participant explained that the students had told her: “But Madame, this is not what happened in the other class. If I did this in the other class, it would be okay.” (Participant 3).

An interesting observation referred to the practice of “assessment bunching.” Upon probing, this referred to inefficiencies in the program design, where assessments were not evenly spaced. When too many assessments are “bunched” at the end of a term, then: “the environment for those types of situations would magnify these types of problems, where the students would have to look for ways to manage overwhelming deadlines.” (Academic Participant 3).

Poor time management by students, as well as peer pressure, were also presented as factors within the people theme. When students are pressed for time, their temptation to engage in dishonest practices increases. Interestingly, this was referred to as “general student behavior, which is not unique to Mauritius.” Peer pressure was aptly described within the professional staff focus group.

If your friends are doing so, what is the problem? … It does not matter if everyone is doing it; it might still be wrong. And that is something that we have got to get over. Students battle. Students to work hard and maybe do not get the marks that they would want, then they see their friends cheating and get good marks. (Professional Participant 5)

Finally, the differences in the values were presented as a complicating factor. A specific reference was made to how integrity, as a virtue, is lost in how children are raised:

If they are being raised just by mobile phones, just by Tiktok. Not having the parents with them, the mother saying, “you should not do that” then these values are not inculcated since childhood, then when they are adults, young adults, they don’t have those values, and then we talk about academic integrity, they will listen, but when they really reach that point where they have to make a decision, they will just go for the easiest way, which is cheating. (Participant 3)

Contextual differences are closely linked to the values. I was not surprised that the differences between how things are done in the UK and Mauritius were presented as a complicating factor:

you have this kind of difference between what the UK value system is and what is done in developing countries, because most of our students come from schools across Africa, Mauritius, I do not know, India, Pakistan whatever. So, there it is quite different from what we consider the values… my program is to teach teachers and sometimes, you know, the teachers themselves, also, because they do not teach that way of writing. The assignments that they would come, without realizing that it is actually not the UK way of doing things, this is the way that they do it here. This is the way that you write, and it is not out of the intention to cheat. It is literally the way that they have always been doing things. So, I think there is something that needs to be realized. It’s okay, we have all these policies, but in the context that we are in, there is a lot of education, especially with many of these skill sets in other countries they would have had these types of ideas instilled in them, right from the school level. (Participant 6)

The cultural background of the students clearly influences their understanding of academic integrity. As seen in the above quotation, students from different educational systems may not have been taught the same values regarding plagiarism and collaboration, leading to misunderstandings of what constitutes academic misconduct. There also appears to be a fine link between collaboration and collusion. Again, this was not an unexpected finding, as Davis (2023) reported similar results in another study. Teamwork is encouraged during the semester but is not permitted during examinations.

To conclude on the factors that complicate academic integrity practices, feedback from both focus groups affirms that academic integrity is necessary, should be applied consistently, and is a moral value that must be inculcated. It is also a skill that develops over time.

The value of academic research is recognized when translated into practice. The following section offers recommendations for policymakers and university managers as well as recommendations for further research.

### Recommendations for practitioners and researchers

Most policies are currently silent on the acceptable use of digital tools in academic institutions, including my own. Given the rise of large language models, policymakers need to revise student conduct and academic integrity policies to incorporate AI. Moreover, Abuadas and Albikawi (2025) affirmed the potential of integrating AI into education, underscoring the importance of including the responsible use of AI in institutional policies and practices. This recommendation aligns not only with the findings of this research but is also supported by the recommendations of Perkins and Roe (2024).

At the case institution, academic integrity awareness events as well as a non-credit-bearing module on academic integrity are examples of practices already in use. These practices should be expanded to broader contexts, and it is recommended that students, staff, and other stakeholders have clear and easy access to academic integrity policies and procedures. Moreover, the consistent application and enforcement of such policies should be strengthened, which requires running academic integrity awareness campaigns for staff and stakeholders (beyond just students). Training in academic integrity policies and practices should be included in the onboarding process for new staff, both academic and professional.

Recognizing that academic integrity is a developable skill, institutional decision makers should also play a role in establishing support systems for students, such as writing centers and tutoring services, to help students strengthen their academic skills. These support systems should include feedback mechanisms through which students can provide input for academic integrity policies. Such mechanisms can identify areas of confusion and serve as a means of engagement and continuous improvement.

“Assessment bunching” was identified as a complicating factor which can be addressed through more strategic academic planning and scheduling. In conjunction with the use of authentic assessments, which require critical thinking and the application of knowledge rather than memorization of content, this approach can reduce opportunities for cheating and encourage deeper engagement with course content.

Finally, policymakers should recognize and address cultural differences that may affect students’ (and staff’s) understanding of academic integrity. However, this does not mean that universally accepted standards and values should be adjusted. Rather, educational efforts should consider these differences to help bridge gaps in understanding.

Future research should include longitudinal studies on academic integrity practices to track changes in student attitudes and behaviors, which can inform strategies for promoting a culture of integrity. It may also be of interest to conduct research on how academic integrity policies are revised in response to the challenges arising from technological advancements. To identify diverse perceptions of academic integrity, future research should engage staff and students from different cultural backgrounds directly, determine how academic practices are interpreted, and identify the steps needed to ensure a unified understanding. Additional studies could test the effectiveness of academic integrity campaigns or which technological tools are most relevant in developing academic integrity as both skill and value. Finally, as indicated earlier, I was not surprised by the comments on the application of UK policies within the Mauritian context. Further research might explore localization practices. The influence of national culture on how ethics and academic integrity are perceived warrants further investigation.

## Conclusion

Academic integrity is a fundamental ethical principle in education, and upholding it is the responsibility of every member of the academic community. The findings from this research call for holistic approaches to academic integrity by building shared understanding across all stakeholders, and, in the case of this research, across countries. Such a shared understanding ought to be developed through the sharing of experiences and contexts, collegial conversations, ongoing training, and the continuous development of resources, tools, instruments, and institutional commitment.

More energy should be invested in the processes that build cultures of integrity rather than focusing on the opposite, such as cheating, plagiarism, and misconduct. However, there may never be a one-size-fits-all solution, given the cultural and institutional diversity within the global higher education community. However, academic integrity is a skill that can be developed and strengthened. A culture of integrity requires ongoing dialogue, training, and development of resources that support ethical practices in education. Ultimately, the aim should be to cultivate environments where academic integrity is seen not merely as a set of rules to follow but as an integral part of the educational experience that prepares students for ethical conduct in their professional lives. While the concept of academic integrity may seem complicated, as alluded to in the title, it is ultimately a positive values-driven approach to learning. The findings from this research highlight its complex nature, which is still framed negatively, focusing on what not to do rather than promoting it as a positive value and skill.

### Data availability statement

The datasets generated during and/or analysed during the current study are available from the corresponding author ( davisa@unisa.ac.za) on reasonable request. The data are not publicly available because of institutional restrictions that could compromise the privacy of the case institution and research participants.

## Ethical approval statement

The research was conducted with ethical clearance approval by the scientific research ethics committee of the College of Economic and Management Sciences within the University of South Africa, certificate number (2022_CRERC_028_FA).

## Data Availability

The data supporting the findings of this study are available upon request from the corresponding author davisa@unisa.ac.za. The data are not publicly available because of institutional restrictions that could compromise the privacy of the case institution and research participants.
