# Supplementary material for: It’s complicated: staff experiences of academic integrity at a Mauritian university
Source: F1000Res. 2026 Jan 9;14:776. Originally published 2025 Aug 8. [Version 2] doi: 10.12688/f1000research.167245.2 (PMC12790601; doi:10.12688/f1000research.167245.2)
Supplement: Peer review history

# Reviewer report: Reviewer response for version 2

**DOI:** 10.5256/f1000research.194954.r448993

**Published:** 2026-01-10

**Authors:** Belle Louis Jinot

**Affiliations:** 1 Open University of Mauritius, Reduit, Mauritius

The revised is fine. It does not require any further changes.

Is the work clearly and accurately presented and does it cite the current literature?

Partly

If applicable, is the statistical analysis and its interpretation appropriate?

Partly

Are all the source data underlying the results available to ensure full reproducibility?

Yes

Is the study design appropriate and is the work technically sound?

Partly

Are the conclusions drawn adequately supported by the results?

Partly

Are sufficient details of methods and analysis provided to allow replication by others?

Yes

Reviewer Expertise:

Education/leadership and management/online teaching and learning

I confirm that I have read this submission and believe that I have an appropriate level of expertise to confirm that it is of an acceptable scientific standard.

# Reviewer report: Reviewer response for version 1

**DOI:** 10.5256/f1000research.184340.r443820

**Published:** 2026-01-06

**Authors:** Martin Oliver

**Affiliations:** 1 UCL Institute of Education, London, England, UK

The article provides an analysis of staff views about academic integrity, comparing and contrasting academic and professional perspectives. This is a welcome approach, which expands previous studies. The empirical site is also under-represented in previous research, making this work distinctive.

 I really appreciated the care taken in describing the research process, and the clarity about positionally. This was handled in an exemplary way.

 Structurally, the order of sections felt a little unusual to me; I would expect positionally to be address in or around the methodology section, and I would normally expect the presentation of themes to be followed by a discussion section relating this to the literature, then conclusions, which would incorporate recommendations. However, all these sections are present in the submitted paper - discussion incorporated with presentation of themes, recommendations preceding conclusions - so I do not think this structure is an issue that needs to be addressed. (Personally, I would also suggest deleting the sentence about the value of research being recognised when translated into practice; I don't think this is needed, and it also doesn't address research that is valued through citation in other research.)

 There are also some minor clarifications that would be worth adding. This includes explaining what Bretag's model consists of, when it's first mentioned; and also, explaining the SADC acronym the first time this is used. I was also slightly confused by one sentence: "The academic staff focus group included six participants from all faculty members.” I wasn't sure if the final clause was intended to signal recruitment from across all disciplines at the institution.

Is the work clearly and accurately presented and does it cite the current literature?

Yes

If applicable, is the statistical analysis and its interpretation appropriate?

Not applicable

Are all the source data underlying the results available to ensure full reproducibility?

No source data required

Is the study design appropriate and is the work technically sound?

Yes

Are the conclusions drawn adequately supported by the results?

Partly

Are sufficient details of methods and analysis provided to allow replication by others?

Yes

Reviewer Expertise:

Higher Education, use of technology, ethics

I confirm that I have read this submission and believe that I have an appropriate level of expertise to confirm that it is of an acceptable scientific standard.

# Response

**Published:** 2026-01-06

**Authors:** Annemarie Davis

**Affiliations:** Business Management, University of South Africa, Pretoria, Gauteng, South Africa

Thank you for taking the time to review the article! I appreciate your input.

 I appreciate the recognition that the empirical site is under-represented – indeed, this is true for many Global South contexts.

 I carefully read through your comments.

 I moved the section on the researcher’s position closer to the methodology, directly following the proposition.

 I deleted the sentence about the value of research being recognised when translated into practice. 

 In terms of the Bretag model, I added detail on the elements and also explained the SADC acronym. 

 On the request for clarification of the academic staff focus group, I amended the sentence to: The academic staff focus group included six participants from all faculties.

 Thank you for the time you invested in reviewing this article!

# Reviewer report: Reviewer response for version 1

**DOI:** 10.5256/f1000research.184340.r439572

**Published:** 2026-01-06

**Authors:** Belle Louis Jinot

**Affiliations:** 1 Open University of Mauritius, Reduit, Mauritius

This study is about the academic staff and supporting staff's perceptions of academic integrity and what factors make academic integrity challenging. It adopts a qualitative research method. However, there is a lack of literature review, particularly on the factors that make academic integrity challenging in Higher Education Institutions. Some of the references are outdated.

 References to the African context are somehow insufficient. The qualitative data interpretation lacks discussion, and it seems too narrative. The use of I makes the study too personal and sometimes the researcher is too judgmental or emotional in the statements (for example, the use of ..I appreciated...).

 There is a lack of ethical consideration as the research site is identified in the section under Discussion.

Is the work clearly and accurately presented and does it cite the current literature?

Partly

If applicable, is the statistical analysis and its interpretation appropriate?

Partly

Are all the source data underlying the results available to ensure full reproducibility?

Yes

Is the study design appropriate and is the work technically sound?

Partly

Are the conclusions drawn adequately supported by the results?

Partly

Are sufficient details of methods and analysis provided to allow replication by others?

Yes

Reviewer Expertise:

Education/leadership and management/online teaching and learning

I confirm that I have read this submission and believe that I have an appropriate level of expertise to confirm that it is of an acceptable scientific standard, however I have significant reservations, as outlined above.

# Response

**Published:** 2026-01-07

**Authors:** Annemarie Davis

**Affiliations:** Business Management, University of South Africa, Pretoria, Gauteng, South Africa

Thank you for taking the time to review the article! I appreciate your input.

 I offer responses to your comments below.

*Reviewer comment: There is a lack of literature review, particularly on the factors that make academic integrity challenging in Higher Education Institutions. Some of the references are outdated. References to the African context are somehow insufficient.*

 Author response: Thank you – I added a paragraph on academic integrity challenges in higher education institutions, with a specific focus on the African context. Specifically, the following:

 Mireku, D. O., Dzamesi, P. D., & Bervell, B. (2024). Plagiarism in Higher Education (PLAGiHE) within Sub-Saharan Africa: A systematic review of a decade (2012–2022) literature. *Research Ethics*, *20*(2), 156-186.

 Mtshweni, B.V., 2024. A theoretical review of contract cheating in master’s and doctoral studies: Some global trends and perspectives. *South African Journal of Higher Education*, *38*(6), pp.119-137.

 Okyere, J., & Ayebeng, C. (2025). Blurring boundaries: Research assistance and the risk of contract cheating in postgraduate education in Africa. *Research Ethics*, 17470161251388118.

 Schoon, A., Smit, A., Uzuegbunam, C., & Bosch, T. (2025). Adoption and perceptions of generative AI among South African academics. *Teaching in Higher Education*, 1-18.

 Ndungu, J. N., & Chepsergon, A. K. (2024). Dual Role of AI in Academic Dishonesty and Integrity Management in the Institutions of Higher Learning in Sub-Saharan Africa. *Journal of Research in Education and Technology*, *2*(1), 69-79.

*Reviewer comment: The qualitative data interpretation lacks discussion, and it seems too narrative. The use of I makes the study too personal and sometimes the researcher is too judgmental or emotional in the statements (for example, the use of ..I appreciated...).*

 Author response: I acknowledge the concern. However, the use of first-person voice in this qualitative report was intentional and methodologically grounded. In qualitative inquiry, the researcher is an integral part of knowledge production, and transparent first-person writing can strengthen reflexivity by making the researcher’s positioning, interpretive role, and analytic decisions visible rather than implied. *I applied the Lichtman (2014) guidance that confirms the use of a reflexive stance (which is often referred to as “confessional” notes) to enhance transparency and credibility in reporting.*

*To address the concern about the manuscript reading as “too personal,” I moved the researcher’s position statement closed to the methodology to clarify how my role, as the researcher and the researcher instrument, was managed to support trustworthiness. I also double checked that the use of the first person narrative style is consistent throughout the article.*

*Lichtman, M. (2014). Qualitative research—a reflexive stance. In Qualitative research—a reflexive stance (pp. 27-52). SAGE Publications, Inc., https://doi.org/10.4135/9781544307756.n2*

*Reviewer comment: There is a lack of ethical consideration as the research site is identified in the section under Discussion.*

 Author response: I note your comment on the identification of the research site. While the institution provided permission to be identified in the research, I now opted to refer to it in generic terms to ensure anonymity.
